# Big data- and machine learning-based analysis of a global pharmacovigilance database enables the discovery of sex-specific differences in the safety profile of dual IL4/IL13 blockade

**DOI:** 10.3389/fphar.2023.1271309

**Published:** 2023-10-26

**Authors:** Kassem Sharif, Mahmud Omar, Adi Lahat, Yonatan Shneor Patt, Howard Amital, Ghanem Zoabi, Nicola Luigi Bragazzi, Abdulla Watad

**Affiliations:** ^1^ Department of Gastroenterology, Sheba Medical Centre, Ramat Gan, Israel; ^2^ Department of Medicine B, Zabludowicz Center for Autoimmune Diseases, Sheba Medical Center, Ramat Gan, Israel; ^3^ Sackler Faculty of Medicine, Tel-Aviv University, Tel Aviv-Yafo, Israel; ^4^ Laboratory for Industrial and Applied Mathematics (LIAM), Department of Mathematics and Statistics, York University, Toronto, ON, Canada; ^5^ Section of Musculoskeletal Disease, NIHR Leeds Musculoskeletal Biomedical Research Unit, Leeds Institute of Molecular Medicine, Chapel Allerton Hospital, University of Leeds, Leeds, United Kingdom

**Keywords:** atopic dermatitis, dual IL4/13 blockade, pharmacovigilance, big data analytics, machine learning, disproportionality analysis, adverse drug reactions, sex medicine

## Abstract

**Background:** Due to its apparent efficacy and safety, dupilumab, a monoclonal antibody that blocks Interleukin 4 (IL-4) and Interleukin 13 (IL-13), has been approved for treating T-helper 2 (Th2) disorders. However, adverse effects like local injection site reactions, conjunctivitis, headaches, and nasopharyngitis have been reported. Sex differences are known to influence both adaptive and innate immune responses and, thus, may have a bearing on the occurrence of these adverse effects. Nevertheless, the literature lacks a comprehensive exploration of this influence, a gap this study aims to bridge.

**Materials and Methods:** A comprehensive data mining of VigiBase, the World Health Organization (WHO) global pharmacovigilance database which contains case safety reports of adverse drug reactions (ADRs) was performed to test for sex -specific safety response to dual IL4/IL13 blockade by dupilumab. The information component (IC), a measure of the disproportionality of ADR occurrence, was evaluated and compared between males and females to identify potential sexual dimorphism.

**Results:** Of the 94,065 ADRs recorded in the WHO global pharmacovigilance database, 2,001 (57.4%) were reported among female dupilumab users, and 1,768 (50.7%) were among males. Immune/autoimmune T-helper 1 (Th1)-, innate- and T-helper 17 (Th17)-driven diseases and degenerative ones were consistently reported with a stronger association with Dupilumab in males than females. Some adverse events were more robustly associated with Dupilumab in females.

**Conclusion:** Dupilumab has an excellent safety profile, even though some ADRs may occur. The risk is higher among male patients, further studies, including *ad hoc* studies, are needed to establish causality.

## Introduction

The human immune system is not “one-size-fits-it-all”, but displays noticeable differences between the sexes (Markle and Fish, 2014; Tokatli et al., 2022). We have defined “sex” as a biological attribute, categorized conventionally as male or female, based on physiological and anatomical distinctions, such as chromosomes, hormone levels, and reproductive/sexual anatomy (Short et al., 2013) This has an impact on various aspects of immunity, including the recognition and components responsible for response, ranging from type 1 to type 3 immunity (Annunziato et al., 2015) and involving both the innate and adaptive systems (Shepherd et al., 2021; vom Steeg and Klein, 2016).

Type 1 immunity protects the body against intracellular microbes by activating mononuclear phagocytes and the oxidative burst. This form of immunity involves T-bet + interferon-gamma (IFN-γ)–producing group 1 innate lymphoid cells or ILCs (ILC1 and natural killer (NK) cells) (Annunziato et al., 2015; Shannon et al., 2021), CD8^+^ cytotoxic T cells (TC1), and CD4^+^ T helper type 1 (Th1) cells. These cells produce and release large quantities of IL-2 and lymphotoxin alpha (LT-α) (Annunziato et al., 2015).

Type 2 immunity is composed of GATA-3+ ILC2s (Zhu, 2017; Spinner and Lazarevic, 2020), T_C_2 cells, and T-helper 2 (T_h_2) cells (Annunziato et al., 2015). Its primary function is to support B-cell production, development, and proliferation, as well as promoting class switching and the release and recruitment of immunoglobulins (Vazquez et al., 2015). Type 2 immunity is crucial in developing helminth infections, allergic/atopic diseases, and airway inflammation observed in asthma patients. This type of immunity is characterized by the molecular signature of IL-4, IL-5 (which stimulate eosinophils and basophils) (Min and Paul, 2008), IL-9, IL-10, and IL-13, along with cytokines produced by epithelial cells such as thymic stromal lymphopoietin (TSLP), IL-25, and IL-33 (Spellberg and Edwards, 2001; Annunziato et al., 2015; Ochiai et al., 2018; Roan et al., 2019).

At the level of the epithelial barrier, type 3 immunity enables defense against pyogenic extracellular bacteria (such as Streptococci and Staphylococci) and fungi. It comprises type 3 ILCs (ILC3), γδ-T cells, CD8^+^ αβ-T cells (T_C_17), and CD4^+^ T_h_17 cells (Short et al., 2013). Group 3 immunity is involved in the recruitment of neutrophils and is characterized by the following molecular signature: IL-17A, IL-17F, and IL-22, along with high amounts of TGF-beta (Annunziato et al., 2015).

Male sex hormones fine-tune cell-mediated immunity, whereas female sex hormones regulate humoral immunity. As a result, males are predisposed toward the Th1 and Th17 milieu, whereas females have a more pronounced/skewered Th2 phenotypic profile (Klein and Flanagan, 2016). However, the precise effects of hormones, such as testosterone, are still not well understood and appear controversial, with some studies, including the pioneering work by Folstad and Karter (Folstad and Karter, 1992), suggesting that testosterone may negatively impact the immune system, exerting pro-oxidant and immunosuppressive activity (the so-called “immunocompetence handicap hypothesis” or “male susceptible hypothesis”) (Stoehr and Kokko, 2006; Nowak-Kornicka et al., 2020). Other studies, on the other hand, have failed to replicate this finding, demonstrating, on the contrary, that testosterone may have immunomodulatory properties.

From a clinical perspective, males are more prone to infectious diseases, particularly more severe phenotypes (such as septicemia/bacteriemia, sepsis, and septic shock) (Annunziato et al., 2015). Females respond better to vaccines but experience a higher incidence of autoimmune disorders (Annunziato et al., 2015).

Atopic dermatitis is a common, relapsing inflammatory skin disease imposing high epidemiological and societal burden, and is characterized by skin barrier impairment, immune dysregulation, and skin dysbiosis. It presents sex-specific differences and worsens during pregnancy (Tuttle et al., 2021).

Limited information exists regarding sexual dimorphism in patients with atopic dermatitis (Tuttle et al., 2021). With the advent of systemic medications like Dupilumab (Dupixent, Regeneron/Sanofi), the pharmacological management of atopic dermatitis as well as other atopic illnesses like asthma and chronic rhinosinusitis with nasal polyps, has undergone a transformation. Dupilumab is a fully-humanized monoclonal IgG4 antibody that functions by blocking IL-4 and IL-13 through the binding of IL-4Rα, a receptor shared by both cytokines (Eichenfield et al., 2022).

Numerous randomized clinical trials (RCTs) have demonstrated the outstanding efficacy and safety profile of Dupilumab. However, it has been suggested that due to sex related differences in both the innate and adaptive immune systems, therapies targeting type 2 immunity, such as Dupilumab, may be more effective in women. Although some RCTs have reported outcomes stratified according to sex, they often neglect to consider a sex based perspective in their discussions of findings, which may result in potential sex bias. This omission could potentially introduce sex bias in clinical conclusions.

With this study, we aim to bridge this gap in knowledge by leveraging data from a global pharmacovigilance database to evaluate the possibility of a sex specific safety response to dual IL4/IL13 blockade by Dupilumab. Our findings may hold potential implications for tailoring treatment strategies to optimize patient outcomes.

## Materials and methods

### Ethical considerations

In VigiBase, case reports maintain the anonymity of both the patient and the reporter. Each case is referenced using a unique national identification number.

### Database

We utilized the global pharmacovigilance database, VigiBase™, developed and maintained by the Uppsala Monitoring Centre (UMC), a Swedish World Health Organization (WHO) Collaborating Centre for International Drug Monitoring. The database contains more than 20 million individual case safety reports (ICSRs) of suspected ADRs that were spontaneously reported by over 140 countries that are part of the WHO Program for International Drug Monitoring, from its inception until 9 March 2021. Although the data is not entirely uniform regarding the relationship between the drug and the reported ADR, it is widely recognized that the comprehensive, data-driven screenings database is crucial for effective pharmacovigilance that can be done quickly.

### Disproportionality analysis

Different measures of disproportionality can be calculated to determine the relationship between a drug and a suspected ADR. These measures include the reporting odds ratio (OR), the proportional reporting ratio (PRR), and the information component (IC). The IC measure, which was initially developed using the Bayesian Confidence Propagation Neural Network (Bate, 2007), indicates the strength of the association between the drug and the ADR. If the lower bound of the IC value is positive (or negative), this means that the drug-ADR pair is reported more often (or less often) than expected, based on all the reports available in VigiBase.

$$IC=\left(\frac{N\;observed+0.5}{N\;expected+0.5}\right)$$

Where

$$N\;expected=\frac{N\;drug*N\;reaction}{N\;total}$$

In this formula, the term “N_expected_” refers to the expected number of case reports for a specific drug-effect pair, while “N_observed_” refers to the actual number of case reports for the same drug-ADR combination being investigated. “N-drug” represents the total number of case reports for the drug being studied, regardless of the adverse effects reported. On the other hand, “N_reaction_” is the number of case reports for the specific adverse effect under study, irrespective of the type of drug used. Lastly, “N_total_” refers to the total number of reports in the database.

IC is considered more statistically robust as it is based on data mining techniques that help to reduce the risk of identifying false statistically significant associations. It can provide a conservative measure of association, which is crucial when dealing with ADRs that have very low expected frequencies obtained from a large database like VigiBase. This feature of IC is essential as it helps to avoid drawing incorrect conclusions from the data, which can have serious implications for public health.

### ADRs Categorization and Classification

The Medical Dictionary for Drug Regulatory Authorities (MeDRA) ontology at the System Organ Class (SOC) level was used to categorize suspected ADRs related to Dupilumab. We chose the MeDRA ontology for its extensive use in pharmacovigilance and its ability to provide detailed information on ADRs.

## Results

The study classified 2,910 probable Dupilumab-related ADR families after analyzing 94,065 ADRs from 37,848 distinct reports in the WHO global pharmacovigilance database. We found that 2,581 (88.7%) of the cases had sex specific information available, with a female-to-male reporting ratio of 1.13:1. Dupilumab-related ADRs reported among females were 2,001 (77.5% of the cases) and those reported among males were 1,768 (68.5% of the cases). Immune/autoimmune (Th1-, innate- and Th17-driven) diseases, as well as degenerative ones, were consistently reported with a stronger association with Dupilumab in males compared with females. Some adverse events were more robustly associated with Dupilumab in females. A few were sex specific and were reported in males or females only.

**FIGURE 1:**
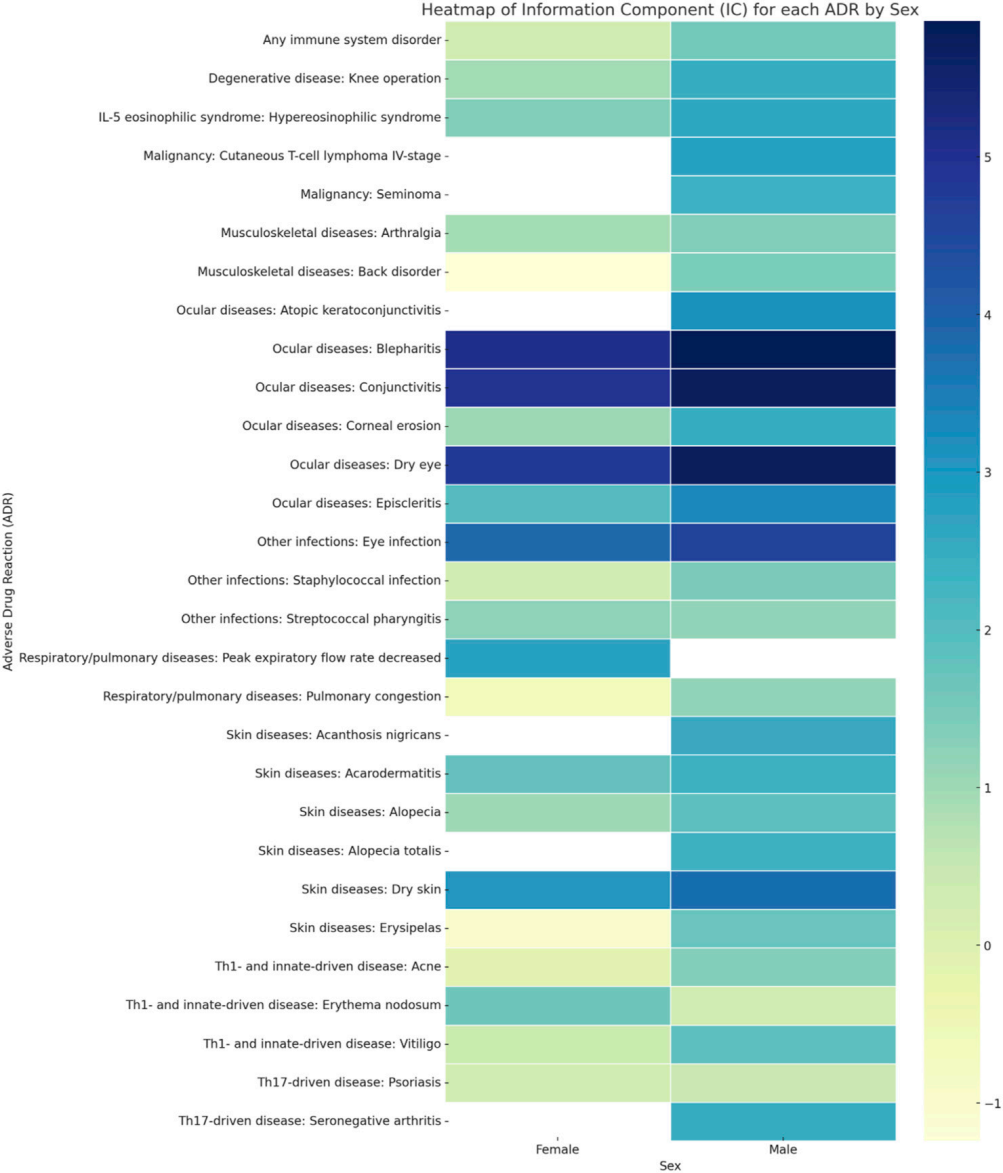
Heat map depicting the information component (IC) of each ADR by sex.

**FIGURE 2:**
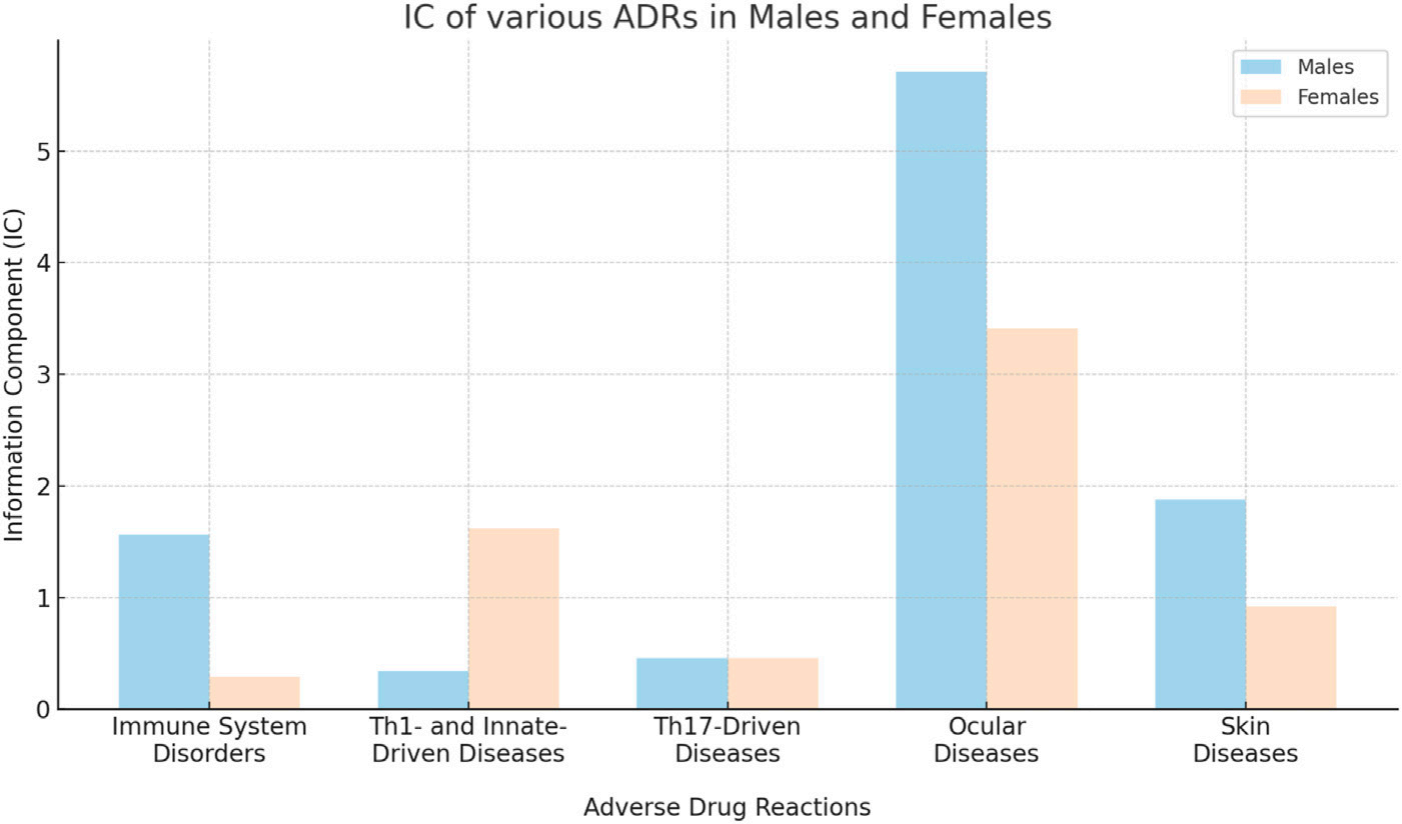
Comparative analysis of information component (IC) for various broad category ADRs in males and females.

**FIGURE 3:**
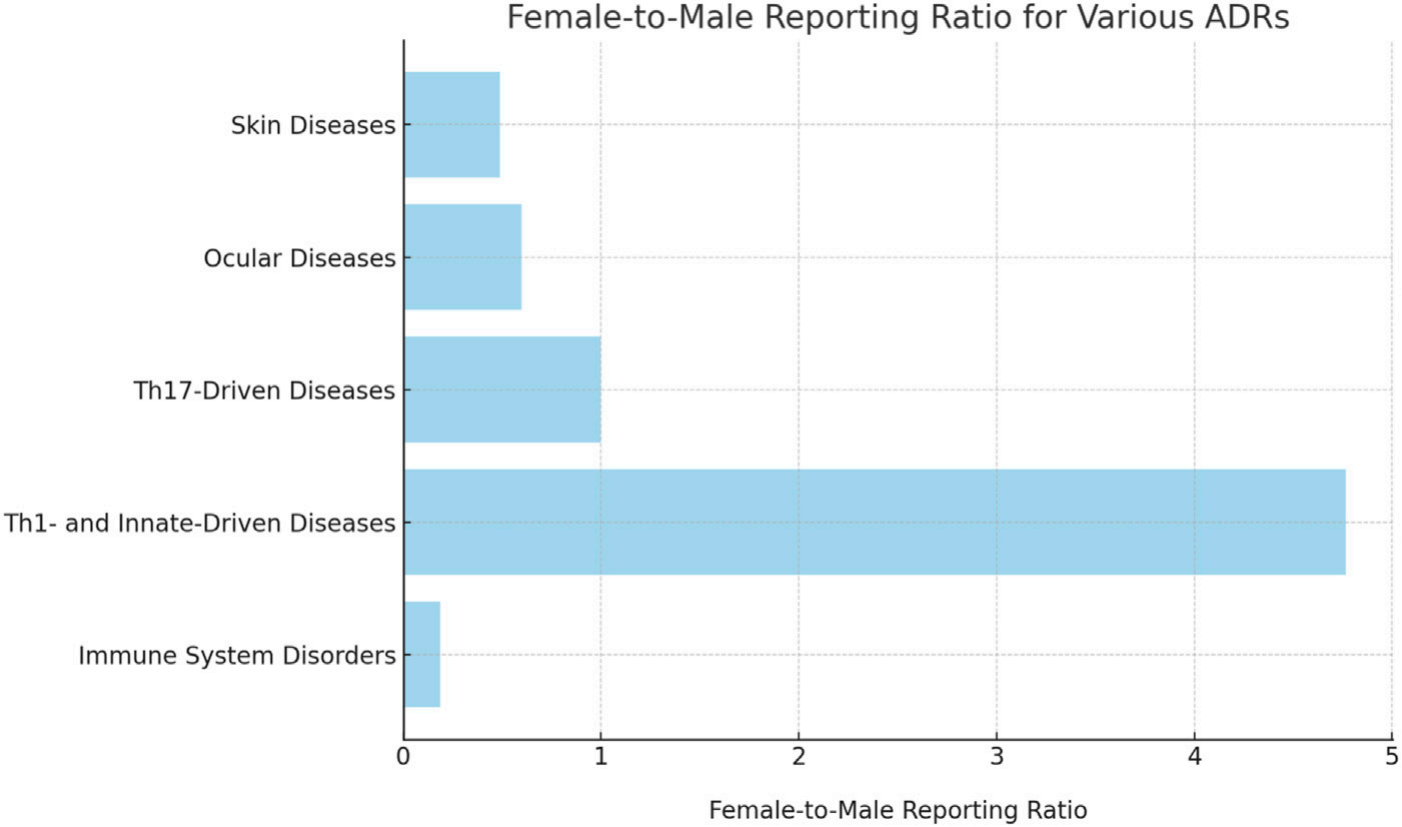
Sex-specific reporting ratio for various broad category ADRs.

**FIGURE 4:**
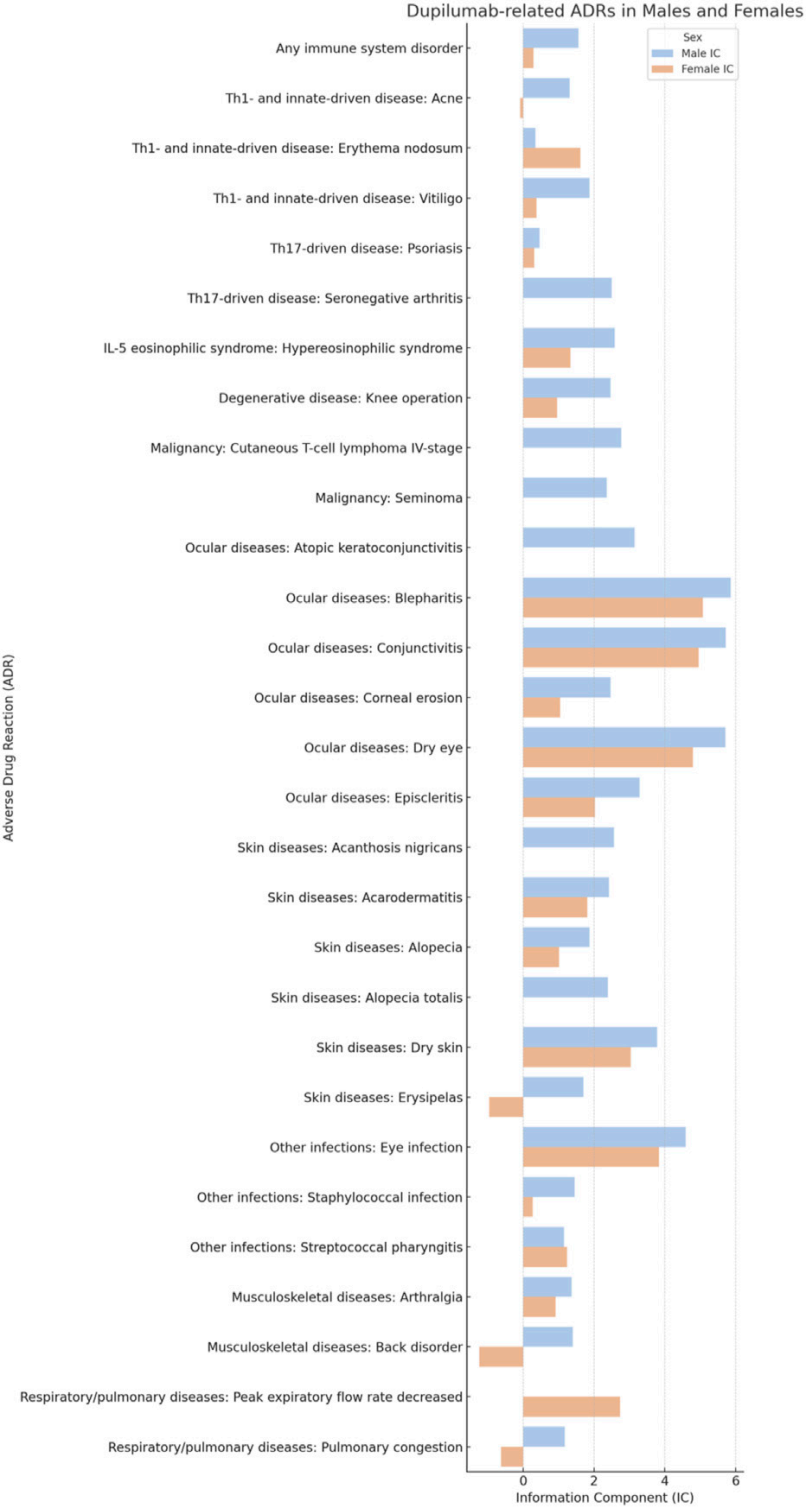
Comparative analysis of specific dupilumab-related adverse drug reactions (ADRs) in males and females.

**FIGURE 5:**
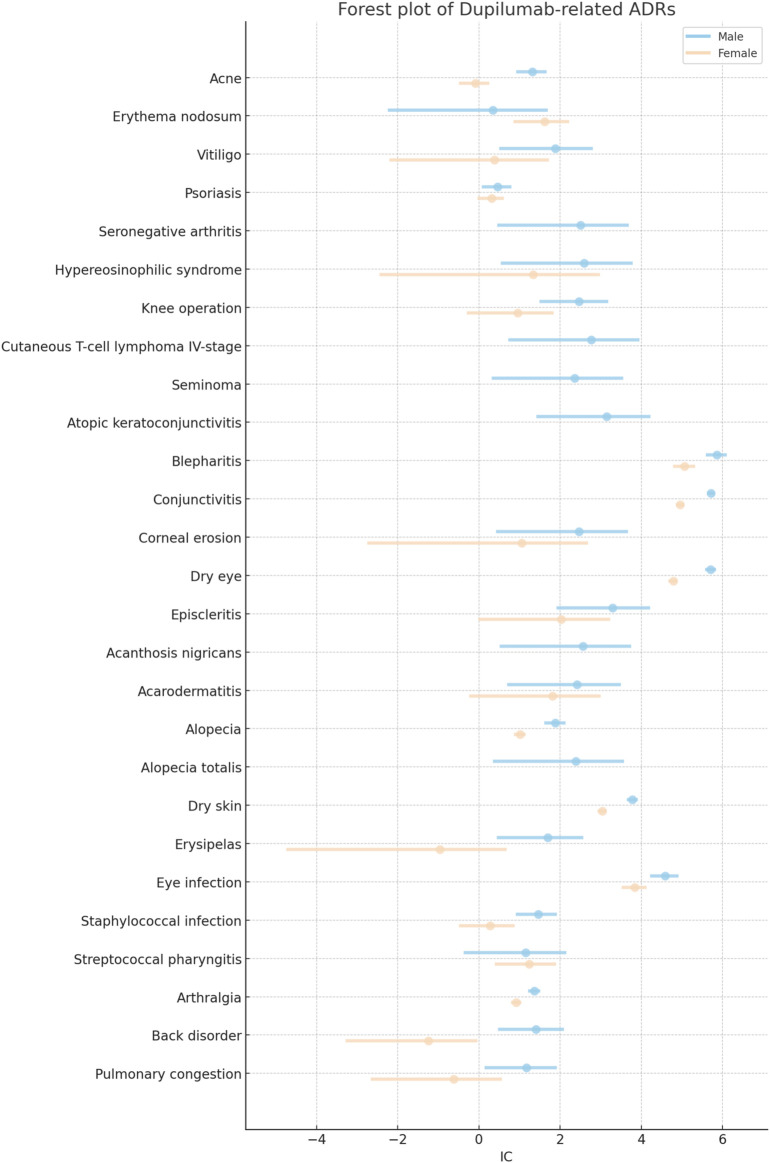
Forest plot of sex-based differences in dupilumab-related ADR.

Table 1 provides a detailed enumeration of these Dupilumab-related ADRs, with Figures 1–5 offering a graphical overview.

**TABLE 1:** Sex-specific dupilumab-related ADRs.

Dupilumab-related ADRs	Male			Female		
	IC	IC025	IC975	IC	IC025	IC975
Any immune system disorder	1.56	0.58	2.28	0.29	−0.87	1.12
*Th1*- and innate-driven disease						
Acne	1.32	0.92	1.67	−0.09	−0.49	0.26
Erythema nodosum	0.34	−2.25	1.70	1.62	0.85	2.22
Vitiligo	1.88	0.50	2.81	0.38	−2.21	1.73
*Th17*-driven disease						
Psoriasis	0.46	0.07	0.80	0.31	−0.04	0.62
Seronegative arthritis	2.50	0.45	3.69	NA	NA	NA
*IL-5* eosinophilic syndrome						
Hypereosinophilic syndrome	2.59	0.54	3.79	1.34	−2.45	2.98
Degenerative disease						
Knee operation	2.47	1.49	3.19	0.96	−0.30	1.84
Malignancy						
Cutaneous T-cell lymphoma IV-stage	2.77	0.72	3.96	NA	NA	NA
Seminoma	2.36	0.31	3.56	NA	NA	NA
Ocular diseases						
Atopic keratoconjunctivitis	3.15	1.41	4.23	NA	NA	NA
Blepharitis	5.86	5.59	6.11	5.07	4.78	5.33
Conjunctivitis	5.72	5.62	5.81	4.96	4.85	5.06
Corneal erosion	2.47	0.42	3.67	1.05	−2.75	2.69
Dry eye	5.71	5.57	5.84	4.79	4.67	4.89
Episcleritis	3.29	1.91	4.22	2.03	−0.02	3.23
Skin diseases						
Acanthosis nigricans	2.56	0.51	3.75	NA	NA	NA
Acarodermatitis	2.42	0.69	3.50	1.81	−0.24	3.003
Alopecia	1.88	1.61	2.13	1.01	0.86	1.15
Alopecia totalis	2.39	0.34	3.58	NA	NA	NA
Dry skin	3.78	3.64	3.92	3.04	2.92	3.15
Erysipelas	1.70	0.44	2.57	−0.96	−4.75	0.68
Other infections						
Eye infection	4.59	4.22	4.92	3.84	3.52	4.13
Staphylococcal infection	1.46	0.91	1.92	0.27	−0.49	0.88
Streptococcal pharyngitis	1.15	−0.38	2.15	1.24	0.39	1.90
Musculoskeletal diseases						
Arthralgia	1.37	1.21	1.51	0.92	0.79	1.04
Back disorder	1.40	0.47	2.10	−1.24	−3.29	−0.04
Respiratory/pulmonary diseases						
Peak expiratory flow rate decreased	NA	NA	NA	2.74	1.21	3.73
Pulmonary congestion	1.17	0.14	1.92	−0.62	−2.67	0.57

Within the immune/autoimmune diseases (Th1-, innate- and Th17-driven), males generally showed higher information component (IC) values than females. For example, males showed an IC of 1.56 for any immune system disorder, compared to a lower IC of 0.58 in females (Table 1; Figure 2).

### Other infections

Even if, overall, the risk for any infectious disease did not differ based on sex (IC 0.51 [95%CrI 0.14-0.83] and IC 0.52 [95%CrI 0.22-0.79], in males and females respectively), some specific infections presented sex related differences. Besides the already mentioned communicable disorders, other infections that were significant in males included eye (IC 4.59 [95%CrI 4.22-4.92]) and staphylococcal (IC 1.46 [95%CrI 0.91-1.92]) infections. Streptococcal pharyngitis was, instead, more strongly associated with (Table 1; Figure 3; Figure 4).

### Malignancies

Dupilumab administration may favor the progression and the exacerbation of cutaneous T-cell lymphoma. In particular, the risk for cutaneous T-cell lymphoma stage IV was found to be increased in males (IC 2.77 [95%CrI 0.72-3.96]). No sex based differences could be found concerning the other stages. Stage I was not statistically associated with Dupilumab both in males (IC 1.54 [95%CrI −2.26 to 3.18]) and females (IC 1.55 [95%CrI −2.25 to 3.19]). Stage II was reported only in females (IC 1.56 [95%CrI −2.24 to 3.20]) but the association was not significant. Stage III was similarly not associated in males (IC 1.58 [95%CrI −2.22 to 3.22]) as well as in females (IC 1.57 [95%CrI −2.23 to 3.21]). Of note, there was a risk for seminoma (IC 2.36 [95%CrI 0.31-3.56]) (Table 1; Figure 4; Figure 5).

### Ocular diseases

Ocular diseases, including dry eye (IC 5.71 [95%CrI 5.57-5.84]), corneal erosion (IC 2.47 [95%CrI 0.42-3.67]), atopic keratoconjunctivitis (IC 3.15 [95%CrI 1.41-4.23]), blepharitis (IC 5.86 [95%CrI 5.59-6.11]), conjunctivitis (IC 5.72 [95%CrI 5.62-5.81]), and episcleritis (IC 3.29 [95%CrI 1.91-4.22]), exhibited higher ICs in males compared to females (Table 1; Figure 1; Figure 4; Figure 5).

### Skin diseases

Alopecia and alopecia totalis, but not alopecia areata (IC 4.09 [95%CrI 3.40-4.64] versus IC 3.41 [95%CrI 2.69-3.99] in males versus females, respectively) were more strongly associated with Dupilumab in males (IC 1.88 [95%CrI 1.61-2.13] and IC 2.39 [95%CrI 0.34-3.58], respectively) (Table 1; Figure 2).

### IL-5 eosinophilic syndrome

Hypereosinophilic syndrome was significantly associated with Dupilumab administration among males (IC 2.59 [0.54-3.79]) but not in females (IC 1.34 [-2.45 to 2.98]).

### Degenerative diseases

Only the need for undergoing knee operation was significantly associated with Dupilumab in males (IC 2.47 [95%CrI 1.49-3.19]). Other degenerative diseases, including cataract (IC 1.17 [95%CrI 0.60-1.65] versus 0.72 [95%CrI 0.25-1.12] in males and females, respectively) and keratoconus, did not differ stratifying according to sex.

In the respiratory disease category, decreased peak expiratory flow rate stood out in females, with an IC of 2.74, whereas this adverse event was not reported in males (Table 1; Figure 4; Figure 5). Hyposmia was another condition statistically significantly related to Dupilumab use only in females (Table 1; Figure 3).

The sex-specific associations are visually interpreted in a heat map (Figure 1) and in Figure 2, Figure 4, and Figure 5, while the female-to-male reporting ratio for various ADR categories provides a comparative view (Figure 3). These results supply valuable insights into sex specific ADRs associated with Dupilumab.

## Discussion

Despite its importance, sex-based medicine is generally overlooked both in research and clinical practice. There is a lack of data concerning the impact of sex on dupilumab-related adverse events. It was observed that adverse drug incidents have a stronger association with Dupilumab in males, despite identifying a female-to-male reporting ratio of 1.13:1, a proportion that is coherent and anticipated as per existing literature (Brabete et al., 2022). We also found that some ADRs were sex-specific, being reported in males or females only.

The finding of a statistically significant association between Dupilumab use and progression/exacerbation of cutaneous T-cell lymphoma may sound surprising and contradict the literature, in that IL4 and IL13 are overexpressed in this malignancy and their dual suppression should inhibit the tumor. It has been, indeed, hypothesized that Dupilumab may be utilized against these types of neoplasm. On the other hand, IL17 has been found to be upregulated in cutaneous T-cell lymphoma and may play a key role in its etiopathogenesis, along with IL23 (Krejsgaard et al., 2013). Another plausible explanation could be an initial misdiagnosis of atopic dermatitis, which exhibits symptoms to those seen in cutaneous T-cell malignancies.

Studies have revealed that sex differences have an impact on various aspects of biologic therapies. For instance, male patients exhibited a better baseline profile than female patients in a large cohort of psoriatic arthritis patients who started TNF inhibitor as their first medication. They had fewer comorbidities, and were more likely to respond to treatment within 3- and 6-month, as well as maintain the treatment for longer periods of time (Højgaard et al., 2018). In contrast, research has shown that females with axial spondyloarthritis have lower response rates and reduced chances of achieving a 12-week response to disease-modifying drugs compared to males (van der Horst-Bruinsma et al., 2013).

The differences between males and females are not only evident in biologic therapies, but also in other treatments like checkpoint inhibitors for cancer. Research shows that in meta-analyses of phase II and III trials of checkpoint inhibitors, both overall survival and progression-free survival improve in both males and females who receive these inhibitors. However, the improvement is significantly greater in males than females for several cancers, such as melanoma, urothelial, and non-small-cell lung cancer (Conforti et al., 2018). In another meta-analysis focusing only on phase III trials, the positive effects of checkpoint inhibitors on overall survival and progression-free survival were more pronounced in males than females. Additionally, male-biased outcomes are more evident in anti-CTLA-4 therapies than in anti-PD-1/PD-L1 therapies (Grassadonia et al., 2018).

Both the innate and adaptive immune responses exhibit sex-based variations. Research has demonstrated that when compared to their male counterparts, females often display larger counts of resting and activating CD4^+^ T cells, CD19^+^ B cells, as well as higher levels of several immunoglobulins, specifically IgE, IgG, and IgM. Additionally, female participants tend to produce more interleukin (IL)-4 and IL-10 in response to phytohemagglutinin-induced polyclonal activation (Girón-González et al., 2000). These variations are assumed to result from acquired (i.e., hormonal) and hereditary causes. Given the above observations, one could hypothesize that therapies targeting type 2 immunity (such as dupilumab, tralokinumab, lebrikizumab, and nemolizumab) may be more effective in females than males. However, it is important to note that there is currently no published research evaluating the clinical efficacy of these drugs in female patients while controlling for differences in pharmacokinetics.

Concerning the mechanisms specific to ADRs and sex discrepancies, both Tralokinumab and Lebrikizumab, monoclonal antibodies targeting IL-13, have been demonstrated to correlate with elevated risks of conjunctivitis as discerned in phase 2 and 3 clinical trials (Simpson et al., 2018; Wollenberg et al., 2019). Given that androgens, predominantly in males, exhibit distinct interactions with T cells, moderating the synthesis of IL-4 and IL-13 while augmenting the expression of Foxp3, this could elucidate why Dupilumab is more robustly associated with ocular conditions in males. Moreover, the hormones estrogen and progesterone, prevalent in females, significantly influence immune responses, with estrogen fostering a TH2 and T regulatory phenotype and progesterone encouraging a TH2 phenotype and the transformation of fetal T lymphocytes into T regulatory cells. This could clarify why, within immune/autoimmune diseases (Th1-, innate- and Th17-driven), males generally exhibit elevated information component (IC) values than females in conditions like Erythema Nodosum and Seronegative Arthritis."

Dupilumab has not been investigated for its effectiveness or safety in pregnant women. Since dupilumab is a recombinant IgG4 monoclonal antibody and is anticipated to have a high intrauterine exposure starting about mid-gestation (Koren and Ornoy, 2018), it is advised that clinicians refrain from prescribing dupilumab to women who are pregnant, want to become pregnant, or are breastfeeding. However, there have been a few cases of pregnant women with atopic dermatitis who have used dupilumab without any reported negative effects on either the mother or the baby (Mian et al., 2020; Lobo et al., 2021).

Sex is a critical biological variable that needs to be considered in subsequent clinical trials involving biological drugs. In a study exploring sex bias in clinical trials in patients with severe asthma, studies involving omalizumab, benralizumab, reslizumab, mepolizumab and dupilumab in severe asthma was higher (60.4%) than the percentage of men. While sex bias in recruitment was not apparent, the separate analysis by sex of the main variable was carried out in only 5 of the 37 studies included, only 1 of the 37 trials discussed results separated by sex and no study included the concept of gender in the text (Ciudad-Gutiérrez et al., 2021).

This study has notable strengths, such as analyzing a significant number of individual case safety reports and utilizing disproportionality measures to thoroughly evaluate drug-ADR associations. Additionally, Our sample aligns with, and is even more comprehensive than, other studies exploring similar subjects and employing analogous methodologies (Khamisy-Farah et al., 2021; Park et al., 2021).

Nevertheless, the study does possess limitations that need recognition. For example, sex bias in the reporting of adverse drug reactions is a prevalent issue in pharmacovigilance studies, and in our particular case, may be influenced by alterations during the menstrual cycle, or age and ensuing hormonal variations. Furthermore, the diverse database sources could introduce bias that might affect the generalizability of our results. The establishment of a direct causal relationship between Dupilumab use and certain ADRs requires further epidemiological surveys and clinical assessments. Previous data attests to the validity and verification of case reports that are published in VigiBase.

In conclusion, our findings underscore the importance of a personalized approach to Dupilumab therapy despite its excellent safety profile, especially considering the sex-specific differences in adverse drug reactions. This provides an impetus for further research aimed at understanding the implications of such differences in a clinical setting.

## Key summary

This study examines the sex-specific safety responses to Dupilumab, an IL-4 and IL-13 blocking monoclonal antibody, by analyzing case safety reports from the WHO global pharmacovigilance database. The results reveal a higher incidence of ADRs with Dupilumab in males, particularly in the context of immune/autoimmune and degenerative diseases, although some ADRs were more robustly associated with females. This underscores the need for further research to establish causality and inform better patient care.

## Data Availability

Publicly available datasets were analyzed in this study. This data can be found here: VigiBase.
